# Affective Primacy vs. Cognitive Primacy: Dissolving the Debate

**DOI:** 10.3389/fpsyg.2012.00243

**Published:** 2012-07-17

**Authors:** Vicky Tzuyin Lai, Peter Hagoort, Daniel Casasanto

**Affiliations:** ^1^Neurobiology of Language Department, Max Planck Institute for PsycholinguisticsNijmegen, Netherlands; ^2^Neurocognition of Language Group, Donders Center for Cognitive NeuroimagingNijmegen, Netherlands; ^3^Department of Psychology, The New School for Social ResearchNew York, NY, USA

**Keywords:** affective primacy, cognitive primacy, context, emotion, task set inertia, words, scene perception

## Abstract

When people see a snake, they are likely to activate both affective information (e.g., dangerous) and non-affective information about its ontological category (e.g., animal). According to the Affective Primacy Hypothesis, the affective information has priority, and its activation can precede identification of the ontological category of a stimulus. Alternatively, according to the Cognitive Primacy Hypothesis, perceivers must know what they are looking at before they can make an affective judgment about it. We propose that neither hypothesis holds at all times. Here we show that the relative speed with which affective and non-affective information gets activated by pictures and words depends upon the contexts in which stimuli are processed. Results illustrate that the question of whether affective information has processing priority over ontological information (or vice versa) is ill-posed. Rather than seeking to resolve the debate over Cognitive vs. Affective Primacy in favor of one hypothesis or the other, a more productive goal may be to determine the factors that cause affective information to have processing priority in some circumstances and ontological information in others. Our findings support a view of the mind according to which words and pictures activate different neurocognitive representations every time they are processed, the specifics of which are co-determined by the stimuli themselves and the contexts in which they occur.

## Introduction

If a person is hiking in a jungle and encounters an unknown creature, affective information associated with the creature might demand immediate evaluation (e.g., is it safe or dangerous?). If a person is taking a biology exam, however, and has to classify animals according to their ontological categories, they might not even notice how dangerous some animals can be. For decades, researchers have debated whether affective or non-affective “cognitive” information gets activated first (Storbeck and Clore, 2007, for review). According to the Affective Primacy Hypothesis (Zajonc, 1980, 2000; LeDoux, 1996), information relevant for affective responses can be activated quickly and automatically, before information about ontological kinds. By contrast, according to the Cognitive Primacy Hypothesis (Lazarus, 1984; Storbeck et al., 2006) perceivers must determine the ontological category of a stimulus before they can evaluate its affective content.

The present study investigates whether one kind of information is always activated faster than the other, or whether the speed with which affective and non-affective information gets activated varies with context. We propose that neither Affective Primacy nor Cognitive Primacy should hold at all times. The stimuli themselves should not fully determine the relative primacy with which affective and cognitive/non-affective information gets activated, nor should the judgments that people make on the stimuli. Rather, the relative speed with which affective and non-affective information gets activated should depend on the context in which stimuli are processed, even when the stimuli themselves and the judgments people make on them are held constant.

Numerous studies have been interpreted as support for either Affective Primacy or Cognitive Primacy. However, the majority of these studies only demonstrate processing of one kind of information or the other. That is, they either demonstrate how rapidly affective information can be activated (Murphy and Zajonc, 1993; LeDoux, 1996) or how rapidly non-affective information can be activated (Clore and Storbeck, 2006; see also Whalen et al., 1998; Sereno et al., 2003; Kirchner and Thorpe, 2006; Kissler et al., 2007; Schupp et al., 2007). But these studies do not directly compare the time courses of affective and non-affective processing *for the same stimuli*, and therefore do not bear directly on the question of which kind of information has processing priority (cf., Weaver et al., 2010). Likewise, a growing number of studies have explored interactions of affective and non-affective cognitive processing (Gray, 2004; Eastwood and Smilek, 2005; Frischen et al., 2008; Pessoa, 2009; Huang et al., 2011), but they do not bear directly on the question of interest here concerning the relative speed with which affective and non-affective aspects of stimuli can be processed. Several studies have directly compared effects of affective priming and non-affective “semantic” priming (e.g., Kunst-Wilson and Zajonc, 1980; Klauer and Musch, 2002; Storbeck and Robinson, 2004). These studies are informative about the relative strength of affective and non-affective priming, but are not informative about the relative speed with which affective vs. non-affective information can be activated when stimuli are processed, which is the question at issue in the debate over Affective vs. Cognitive Primacy.

In one notable exception, Nummenmaa et al. (2010)conducted a series of experiments designed to directly compare the speed of affective and non-affective judgments for complex scenes. Their results appear robust: across six experiments, non-affective judgments about the ontological category of stimuli were faster than affective judgments about the pleasantness of the stimuli. The authors concluded that, at least when people are processing novel visual scenes, “semantic recognition” of the ontological category of the depicted objects “*must precede* affective evaluation of the scene contents” (p. 242, italics added).

We believe this conclusion is not likely to generalize. The debate over Cognitive Primacy vs. Affective Primacy presupposes that one kind of processing will always (or generally) be faster than the other – at least for certain classes of stimuli, such as complex scenes. Yet, whether affective information gets activated faster or slower than ontological information will surely depend on (1) the particulars of the stimuli and (2) the judgments that people make, and should also depend on (3) the context in which the stimuli are encountered and the judgments are made.

Proposals 1 and 2 can be confirmed in a thought experiment. Even within a given stimulus type such as complex scenes, the relative speed with which people can make affective and ontological judgments should be easy to manipulate. For example, if people are photographed from a distance, identifying their gender should be faster than judging their emotional expressions; for photos that zoom in on people’s facial features, the opposite may be true. Likewise, it should be trivial to manipulate whether an ontological judgment is faster or slower than an affective judgment by making the judgments harder or easier relative to one another, e.g., by manipulating how abstract the judgments are, or how common they are.

The third proposal requires further consideration. Setting aside the issue of whether affective and non-affective stimuli and judgments can ever be meaningfully equated, does the relative speed with which people activate affective vs. ontological information depend on some general principle of how emotion is related to cognition? Or alternatively, does it depend on the context in which the stimuli are being evaluated?

To find out, here we asked participants to make affective and non-affective judgments on stimuli they encountered in two different biasing contexts: an affective context and a non-affective context. Across participants, the same judgments were made on the same stimuli in both of the contexts: only the context varied. To create the contexts, we used a modified “Task Set Inertia” (TSI) paradigm (Allport and Wylie, 2000). Half of the stimuli were words that named humans or animals which were positively or negatively valenced, and the other half were pictures of complex indoor or outdoor scenes which were either pleasant or unpleasant. Words and pictures were randomly intermixed and the type of judgments participants made on them (affective or non-affective) varied orthogonally. As such, word judgments served as a biasing context for picture judgments (Experiment 1), and picture judgments served as a biasing context for word judgments (Experiment 2). Importantly, we made no attempt to equate the “biasing power” of affective vs. non-affective orienting judgments, nor to equate the “bais ability” of affective vs. non-affective target judgments, or of picture vs. word stimulus items. Instead, we constructed a fully within-judgment and within-item design, and predicted an interaction of Context Type × Judgment Type for pictures (in the context of words) and for words (in the context of pictures).

If context determines the relative speed with which affective and non-affective information gets activated – holding both the stimuli and the judgments constant – then affective judgments should be faster in the context of other (irrelevant) affective judgments than in the context of non-affective judgments. Likewise, non-affective judgments should be faster in a non-affective context than in an affective context. Alternatively, if one kind of information is necessarily activated faster than the other, in accord with either the Affective Primacy Hypothesis or the Cognitive Primacy Hypothesis, then either affective or non-affective judgments should be faster, across contexts.

## Experiment 1: Affective and Non-Affective Judgments of Complex Visual Scenes

Experiment 1 tested the context-dependence of affective and non-affective information cued by complex visual scenes (targets), in the context of words (distractors). We predicted a context-congruity effect: the relative speed with which affective and non-affective information could be activated in response to the target pictures should vary according to the type of processing (affective or non-affective) participants were required to perform on the context trials.

### Materials and methods

#### Participants

Thirty-four Native Dutch-speaking undergraduates (mean age = 20.6) at the Radboud University Nijmegen participated with payment. Of these participants, two had low accuracy (<75%) and were excluded. Of the remaining 32, 16 were assigned to the affective context group and 16 to the non-affective context group. Participants gave informed consent before participation.

#### Materials

The stimuli consisted of 96 photos of complex scenes, 24 each of 4 types: pleasant indoor, unpleasant indoor, pleasant outdoor, unpleasant outdoor scenes. In a pretest, two independent raters viewed the pictures, one picture at a time, and judged whether each picture was positive or negative in valence. Inter-coder agreement was 100%, confirming that half of the pictures were clearly positive and the other half clearly negative. The 96 scenes were presented over two blocks. For each participant, 12 of each of the 4 types of nouns were randomly selected to be included in the first block, and the remaining 12 of each type were presented in the second block. The participants made affective judgments (Pleasant/Unpleasant) on the scenes for one of these blocks, and non-affective judgments (Indoor/Outdoor) for the other block. The order of the blocks was counterbalanced between participants.

To create a biasing context, we adapted the TSI paradigm (Allport and Wylie, 2000): randomly intermixed with the target scene trials were an equal number of distractor trials on which participants made either affective or non-affective judgments on words. The words consisted of 96 nouns, 24 each of 4 types: positive-valence animals (e.g., *konijntje* “bunny,” *panda* “panda,” etc.), negative-valence animals (e.g., *parasite*, “parasite,” *kakkerlak*, “cockroach,” etc.), positive-valence humans (e.g., *prinses* “princess,” *grootvader* “grandfather” etc.), and negative-valence humans (*moordenaar* “murderer,” *pedofiel* “pedophile,” etc.)

A norming pretest was carried out to select positive- and negative-valence words. Eighteen native Dutch speakers participated in the pretest for payment. Each participant was given 145 nouns, one word at a time, and was to rate the valence of each noun on a 9-point Self-Assessment Manikins scale (Lang, 1980), ranged from a smiling figure at the positive end of the scale to a frowning one at the negative end (see Jasmin and Casasanto, 2012, for further details). Based on the rating results, we chose 96 nouns that were clearly positive or negative. The mean valence ratings were 6.78 (SD = 0.59) for the positive nouns and 2.79 (SD = 0.82) for the negative nouns. The valence for the two types differed significantly, as confirmed by a two-tailed *t*-test (*t* = 27.29, *p* = 0.0001). Although our within-item design does not require the length, the log frequency, or the arousal of words in different categories to be matched, we still matched these factors.

Participants assigned to the affective context group made affective judgments (positive/negative) for all of the words. Participants assigned to the non-affective context group made non-affective (animal/human) judgments for all words. Thus, the word-judgment trials created either an affective or a non-affective “task set” as the context in which participants made both affective and non-affective judgments on the target scenes.

#### Procedure

Participants sat in a comfortable chair about 90 cm from a monitor in a soundproof, dimly lit experimental booth. Stimuli were presented on a computer monitor (resolution = 1024 × 768 pixels). In the target (scene) trials, the picture was presented for 300 ms, followed by a dark screen until a judgment was made. In the distractor (word) trials, the word was presented for 200 ms, also followed by a dark screen until a judgment was made. Participants were instructed to press the response keys (e.g., indoor, outdoor) as quickly and accurately as possible. Participants responded with the index fingers of both hands, and the left-right positions of keys corresponding to the affective and the non-affective responses were counterbalanced across participants. A brief practice was given at the beginning of the session, and a brief break was given between the two blocks. Each session lasted approximately 15 min.

### Results

**Figure 1:**
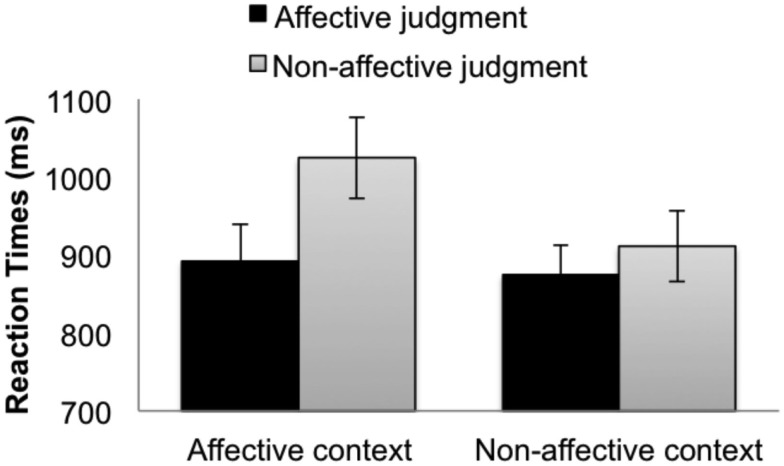
**Reaction times for the (picture) targets when participants made affective judgments (black bars) and non-affective judgments (gray bars) in the affective context group (left bars) and the non-affective context group (right bars)**. The error bars indicate subject-wise SEM.

Inaccurate trials were excluded, resulting in the removal of 9.26% of the data. We planned to remove RTs greater than 5000 ms, but for all trials RTs were less than 5000 ms. The mean RTs from the correct responses for the target trials are summarized in Figure 1.

To test the predicted effect of context on accuracy and RTs, we carried out linear mixed-effects regression models of two context types (affective, non-affective) × 2 judgment types (affective, non-affective), using the lmer4 package in R. Following the guidelines in Baayen (2008), we centered the means. We also employed the principle of forward selection to construct two statistical models (Table 1): a simpler model that took into consideration the random intercept of subject and items (RI model), and a more conservative model which took into consideration not only the random intercept of subject and item but also the random slopes of subject and/or item whenever it was appropriate (i.e., when the factor is a within-subject factor (RIS model). To interpret the significance, we adopted the criterion that a given coefficient is significant if the absolute value of *t* exceeds 2 (Baayen, 2008).

**Table 1:** **The linear mixed-effects regression models on the reaction times for the (picture) targets**.

Exp 1: pictures-as-target, RTs	Coefficient	SE	*t*	Random slope
**RI MODEL**				
Intercept	926.05	29.41	31.49*	
Judgment type	41.74	5.55	7.52*	
Context type	−31.52	28.67	−1.10	
Context type × judgment type	−24.57	5.56	−4.42*	
**RIS MODEL**				
Intercept	927.17	29.45	31.49*	
Judgment type	41.58	14.91	2.79*	Sub, item
Context type	−32.26	28.77	−1.12	Item
Context type × judgment type	−24.45	14.63	−1.67	Sub, item

*A coefficient is a significant predictor of reaction time if |*t*| > 2.

*RI Model includes the random intercept of subject and items. RIS Model includes both the random intercept of subject and items and the random slopes of subject and/or item when the factor is a within-subject factor*.

#### Accuracy

The mean accuracy for all target trials was 90.8% (SE = 0.9%). Within the affective context group, accuracy was 92.2% (SE = 1.3%) for affective judgments and 92.0% (SE = 1.5%) for non-affective judgments. Within the non-affective context group, accuracy was 90.7% (SE = 1.5%) for affective judgments and 88.9% (SE = 1.8%) for non-affective judgments. There were no interactions of main effects of context type or judgment type, according to the RI and RIS models.

#### Reaction times

For RTs, the context type interacted significantly with the judgment type according to the RI model (Table 1, top). This interaction was anon-significant trend in the RIS model (Table 1, bottom). There was a main effect of judgment type, indicating that affective judgments were made faster than non-affective judgments. Planned comparisons within groups indicated that affective responses were significantly faster than non-affective responses in the affective context group (Figure 1, left bars), by both the RI model (Coefficient = 66.01, SE = 7.78, *t* = 8.49) and the RIS model (Coefficient = 65.82, SE = 21.78, *t* = 3.02). In the non-affective context group (Figure 1, right bars), affective responses were significantly faster than non-affective responses in RI model (Coefficient = 18.12, SE = 8.03, *t* = 2.27), but this difference was not significant in the RIS model (Coefficient = 18.11, SE = 20.06, *t* = 0.90). Between groups, no pairwise differences were significant.

As predicted, the RT advantage for affective judgments relative to the non-affective judgments was significantly greater in the affective context group (132 ms) than in the non-affective context group (36 ms) according to the RI model. This interaction suggests that context modulates the relative speed with which affective and non-affective information is activated, even when the judgments people make and the stimuli on which they make them are held constant.^1^ The finding that this interaction was significant according to the RI model, but not according to the more conservative RIS model, suggests that this effect should be interpreted with caution.

Importantly, the effect of context cannot be attributed to superficial similarities between the responses participants made during “context” and “target” trials. This is evident from an examination of the pairwise comparisons that drove the predicted interaction. If the effect of context had only been present for the affective judgments, this would support a possible skeptical explanation of these effects: perhaps making “positive/negative” judgments on words during distractor trials facilitated making “pleasant/unpleasant” judgments on target trials due to the semantic similarity of “positivity” and “pleasantness.” Yet, contrary to this skeptical possibility, the effect of context was not driven primarily by the affective condition (compare dark bars, Figure 1), but rather by the non-affective condition (compare light bars, Figure 1). The effect of context was found most strongly within ontological category judgments, which were superficially dissimilar between the distractors and the targets (e.g., *human-animal* judgments facilitated *indoor-outdoor* judgments). Since the effect of context cannot be explained away by superficial similarities between distractor and target judgments, we suggest that the biasing context influenced RTs by orienting participants toward the relevant dimension of the target stimulus (i.e., its affective content or ontological category), consistent with the orienting effects found in previous TSI experiments (Allport and Wylie, 2000; Brookshire et al., 2010).

The main effect of judgment type (affective vs. non-affective), which was significant according to both the RI and RIS models, bears most directly on the claim made by Nummenmaa et al. (2010) regarding Affective vs. Cognitive Primacy. Nummenmaa and colleagues concluded that non-affective judgments about the ontological categories of stimuli necessarily precede affective judgments – at least for judgments of complex visual scenes – consistent with the Cognitive Primacy Hypothesis. Contrary to their findings, we show here that affective judgments preceded ontological category judgments of complex visual scenes. Since the present data show the opposite pattern from Nummenmaa et al. (2010), clearly it is not the case that one kind of judgments “must precede” the other kind, as a general rule.

## Experiment 2: Affective and Non-Affective Judgments of Words

Experiment 2 tested whether the relationship between affective and non-affective ontological processing is also context-dependent for verbal stimuli, using a “mirror” version of Experiment 1. The words were now used as the target trials, and the scenes as the distractor trials, which provided an affective context for half of the participants and a non-affective context for the other half. We predicted that the effect of congruity between the type of context and the type of target judgments should be found for verbal stimuli.

### Materials and methods

#### Participants

Thirty-five Native Dutch-speaking undergraduates (mean age = 21.4) at the Raboud University Nijmegen participated in this experiment for payment. None of them previously took part in Experiment 1. Of these participants, three had low accuracy (<75%) and were excluded. Of the remaining 32, 16 were assigned to the affective context group and 16 to the non-affective context group. Participants gave informed consent before participation.

#### Materials

The materials and the procedure were the same as in Experiment 1, with the following exceptions. The roles of the scenes and words in the experimental design were reversed, so that the words became the targets and the scenes the distractors. Target judgments on the words were manipulated within subjects (i.e., they performed “positive/negative” judgments for one block of trials and “animal/human” judgments for the other, with block order counterbalanced across subjects). Distractor judgments on the scenes were manipulated between subjects, providing each participant with either an affective or a non-affective biasing context: half of the participants performed “positive/negative” judgments on the scenes, and the other half performed “indoor/outdoor” judgments.

### Results

**Figure 2:**
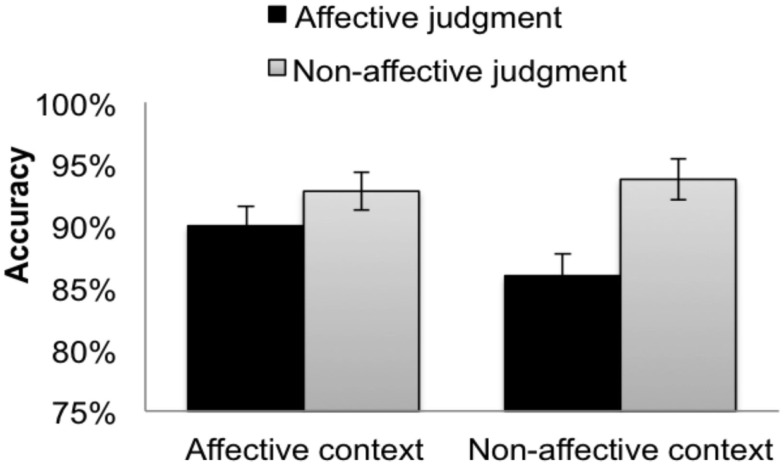
**Accuracies for the (word) targets when participants made affective judgments (dark bars) and non-affective judgments (light bars) in the affective context group (left bars) and the non-affective context group (right bars)**. The error bars indicate subject-wise SEM.

Inaccurate trials were excluded, resulting in the removal of 9.56% of the data. RTs > 5000 ms were also excluded, resulting in the removal of 0.03% of the remaining data. The mean RTs from the correct responses for the target trials are summarized in Figure 2.

To test the predicted effect of context on Accuracy and RTs, we carried out linear mixed-effects regression models of two context types (affective, non-affective) × 2 judgment types (affective, non-affective), using the lmer4 package in R as in Experiment 1 (Tables 2 and 3).

**Table 2:** **The linear mixed-effects regression models on the accuracies for the (word) targets**.

Exp 2: words-as-target, accuracy	Coefficient	SE	*t*	Random slope
**RI MODEL**				
Intercept	0.90	0.02	45.20*	
Judgment type	0.03	0.004	5.95*	
Context type	−0.01	0.01	−1.19	
Context type × judgment type	0.012	0.004	2.96*	
**RIS MODEL**				
Intercept	0.90	0.02	45.25*	
Judgment type	0.03	0.01	3.50*	Sub, item
Context type	−0.01	0.01	−1.19	Item
Context type × judgment type	0.012	0.01	2.02*	sub, item

*A coefficient is a significant predictor of reaction time if |*t*| > 2.

**Table 3:** **The linear mixed-effects regression models on the reaction times for the (word) targets**.

Exp 2: words-as-target, RTs	Coefficient	SE	*t*	Random slope
**RI MODEL**				
Intercept	863.07	25.13	34.35*	
Judgment type	−6.71	5.42	−1.24	
Context type	17.58	24.31	0.72	
Context type × judgment type	−30.10	5.38	−5.59*	
**RIS MODEL**				
Intercept	863.50	25.20	34.20*	
Judgment type	−6.00	12.58	−0.48	sub, item
Context type	18.34	24.46	0.75	item
Context type × judgment type	−29.31	12.33	−2.38*	sub, item

*A coefficient is a significant predictor of reaction time if |*t*| > 2.

#### Accuracy

The mean accuracy for all target trials was 90.4% (SE = 0.8%, SD = 4.7%). There was a significant context type × judgment type interaction in both the RI and RIS models, and a significant main effect of the judgment type: non-affective judgments were more accurate than affective judgments (Table 2). Within the non-affective group (Figure 2, right bars), non-affective judgments were more accurate than affective judgments (RI Coefficient = 0.04, SE = 0.01, *t* = 6.23; RIS Coefficient = 0.04, SE = 0.01, *t* = 3.86). Within the affective group (Figure 2, left bars), non-affective judgments were more accurate than affective judgments according to RI (Coefficient = 0.01, SE = 0.01, *t* = 2.33), but not RIS (Coefficient = 0.01, SE = 0.01, *t* = 1.69). Comparing judgments between context groups, affective judgments (Figure 2, black bars) were more accurate in affective context than in non-affective context (Coefficient = −0.04, SE = 0.02, |*t*| = 2.22). No other significant pairwise differences were significant.

#### Reaction times

**Figure 3:**
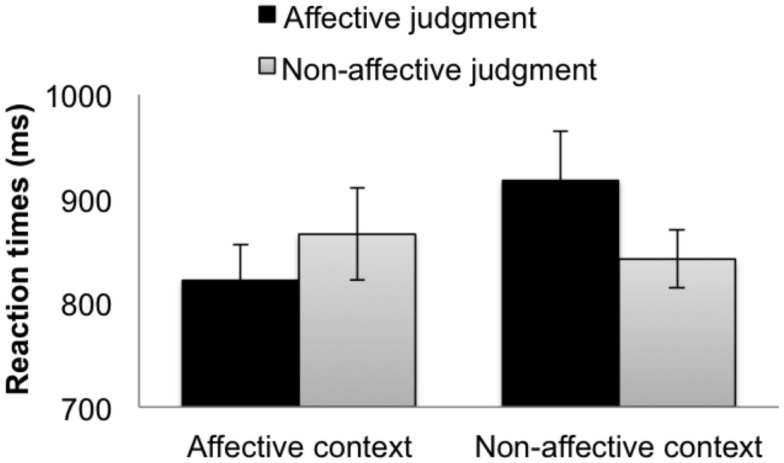
**RTs for the (word) targets when participants made affective judgments (black bars) and non-affective judgments (gray bars) in the affective context group (left bars) and the non-affective context group (right bars)**. The error bars indicate subject-wise SEM.

Significant context type × judgment type interactions were found in both the RI and RIS models (Table 3), suggesting that context modulates the speed of affective and non-affective responses for words. Within the affective context group (Figure 3, left bars), affective judgments were faster than non-affective judgments in the RI model (Coefficient = 22.58, SE = 7.44, *t* = 3.04), but this difference was not significant in the more conservative RIS model (Coefficient = 22.61, SE = 19.33, *t* = 1.17). Conversely, within the non-affective context group (Figure 3, right bars), non-affective judgments were significantly faster than the affective judgments, in both models (RI Coefficient = −36.80, SE = 7.92, |*t*| = 4.65; RIS Coefficient = −35.84, SE = 15.87, |*t*| = 2.26). No other pairwise differences were significant.

Thus, as predicted, the representations cued by words were modulated by context, as reflected by the interaction between the context type and the judgment type in the RTs. In addition, the analysis of accuracy showed that people were more accurate in making non-affective judgments than affective judgments, and that non-affective accuracy advantage was significantly greater in the non-affective context group (7.9%) than in the non-affective context group (2.8%). Thus, the processing context modulated both the speed and the accuracy of participants’ judgments.

#### Combining experiments 1 and 2

**Figure 4:**
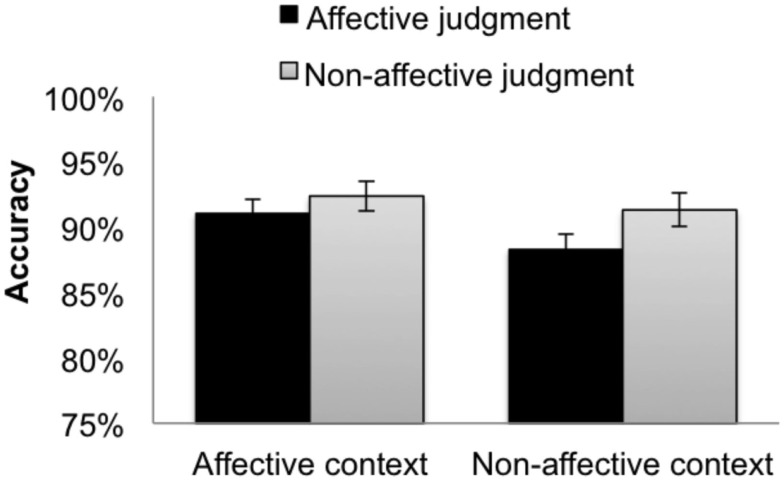
**Accuracies for the combined (picture and word) targets when participants made affective judgments (dark bars) and non-affective judgments (light bars) in the affective context group (left bars) and the non-affective context group (right bars)**. The error bars indicate subject-wise SEM.

**Figure 5:**
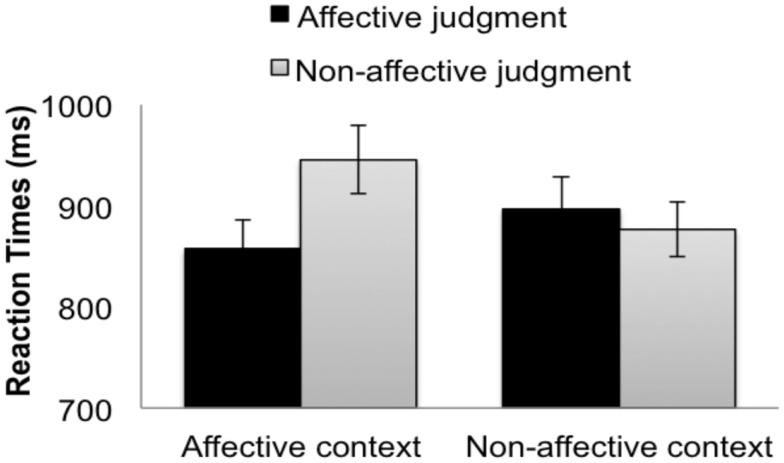
**Reaction times for the combined (picture and word) targets from Experiments 1 and 2 when participants made affective judgments (dark bars) and non-affective judgments (light bars) in the affective context group (left bars) and the non-affective context group (right bars)**. The error bars indicate subject-wise SEM.

A further analysis tested whether the effects of context differed according to the format of the stimuli being judged, pictorial (Experiment 1) or verbal (Experiment 2). The combined Accuracy data is plotted in Figure 4, and the combined RTs in Figure 5. We conducted a linear mixed-effects regression on the results of Experiments 1 and 2 (2 experiments × 2 context types × 2 judgment types).

#### Accuracy

There was a three-way interaction of context type, judgment type, and experiment, as well as a two-way interaction of context type and judgment type (Table 4). There was no experiment by context type interaction, or main effect of experiment. Pairwise comparisons showed that within non-affective context, non-affective judgments were more accurate than affective judgments (RI Coefficient = 0.02, SE = 0.005, *t* = 3.28; RIS Coefficient = 0.02, SE = 0.01, *t* = 2.12). Holding judgment type constant, affective judgments were more accurate in affective context than in non-affective context (RI Coefficient = −0.02, SE = 0.006, |*t*| = 2.46). There were trends in the predicted directions for the remaining pairwise comparisons, but the observed differences were not significant.

**Table 4:** **The linear mixed-effects regression models on the accuracies for the (picture and word) targets**.

Exp 1 + exp 2, accuracy	Coefficient	SE	*t*	Random slope
**RI MODEL**				
Intercept	0.90	0.02	45.63*	
Exp: pictorial or verbal	0.003	0.03	0.12	
Judge: judgment type	0.03	0.004	5.97*	
Context: context type	−0.01	0.01	−1.07	
Exp × judge	−0.03	0.01	−5.42*	
Exp × context	−0.004	0.01	−0.41	
Judge × context	0.01	0.004	2.97*	
Exp × judge × context	−0.02	0.01	−2.58*	
**RIS MODEL**				
Intercept	0.90	0.02	45.66*	
Exp: pictorial or verbal	0.004	0.03	0.15	
Judge: judgment type	0.03	0.01	3.73*	Sub, item
Context: context type	−0.01	0.01	−1.21	Item
Exp × judge	−0.03	0.01	−3.34*	Item
Exp × context	−0.003	0.01	−0.35	Item
Judge × context	0.01	0.01	2.26*	Sub, item
Exp × judge × context	−0.02	0.01	−1.99	Sub, item

*A coefficient is a significant predictor of reaction time if |*t*| > 2.

#### Reaction times

There was no three-way interaction in either RI or RIS models (Table 5). Therefore, there was no significant difference between pictures and words in terms of the strength of the effect of context on target judgments. There was no experiment by context type interaction, and no main effect of experiment. The significant context type × judgment type interaction found in each experiment was replicated in the data from both experiments, combined. Planned comparisons showed that within the affective context, affective judgments were faster than non-affective judgments (RI Coefficient = −44.18, SE = 14.84, |*t*| = 2.98; RIS Coefficient = −44.34, SE = 5.40, |*t*| = 8.22). There were trends in the predicted directions for all other pairwise comparisons, but the observed differences were not significant.

**Table 5:** **The linear mixed-effects regression models on the reaction times for the (picture and word) targets**.

Exp 1 + Exp 2, RTs	Coefficient	SE	*t*	Random slope
**RI MODEL**				
Intercept	863.07	27.35	31.55*	
Exp: pictorial or verbal	63.00	38.68	1.63	
Judge: judgment type	−6.73	5.50	−1.22	
Context: context type	17.58	26.58	0.66	
Exp × judge	48.48	7.76	6.25*	
Exp × context	−49.09	37.59	−1.31	
Judge × context	−30.10	5.46	−5.51*	
Exp × judge × context	−5.53	7.74	0.72	
**RIS MODEL**				
Intercept	863.30	27.39	31.53*	
Exp: pictorial or verbal	63.85	38.72	1.65	
Judge: judgment type	−6.64	13.78	−0.48	Sub, item
Context: context type	17.99	26.66	0.68	Item
Exp × judge	48.59	19.48	2.49*	Item
Exp × context	−50.32	37.70	−1.34	Item
Judge × context	−29.74	13.48	−2.21*	Sub, item
Exp × judge × context	5.37	19.07	0.28	Sub, item

*A coefficient is a significant predictor of reaction time if |*t*| > 2.

## Discussion

In two experiments we show that the context in which stimuli are processed can determine the relative speed with which people make affective and non-affective judgments on pictures and words. The effect of processing context was found even though the stimuli themselves and the judgments participants made were held constant. Context had a moderate effect on the relative speed with which affective and non-affective information was activated in response to complex visual scenes, which was significant according to the simpler of the two statistical models we constructed, but not according to the more conservative model. For judgments on words, the effect of context caused a complete reversal of the relationship between affective and non-affective ontological judgments: affective judgments were faster than non-affective judgments in an affective context, whereas non-affective judgments were faster than affective judgments in a non-affective context. Overall, these findings challenge both the Affective Primacy and the Cognitive Primacy hypotheses, belying any broad generalization about processing priority for affective or ontological information.

The main effect of judgment type that we report for judgments of complex visual scenes (Experiment 1) disconfirms the conclusions of the most direct test of Affective Primacy vs. Cognitive Primacy to date. Whereas non-affective judgments were faster than affective judgments of complex visual scenes in the study by Nummenmaa et al. (2010), the opposite pattern was found for scene judgments here. Even within the test bed of complex visual scenes, there is no consistent answer to the question of whether affective or ontological information gets activated faster.

The question of Affective Primacy vs. Cognitive Primacy, which has been debated for three decades, is ill-posed: there is no unique answer. The time course over which affective and ontological information gets activated may still be fruitfully investigated, so long as the goal of the investigation is not to determine which kind of information gets activated first *as a rule*. A more productive goal may be to determine the factors that cause affective information to have processing priority in some circumstances and ontological information in others. Our suggestion is compatible with a growing movement in the affective and cognitive sciences to consider people’s thoughts and emotions not as fixed representations to be “accessed” from a static memory bank, but rather as representations that are constructed dynamically, activating information in long-term memory *ad hoc*, on the basis of both the proximal cues (e.g., words, pictures) and the physical and social contexts in which they occur (Barsalou, 1985; Eastwood and Smilek, 2005; Barrett, 2006; Frischen et al., 2008; Casasanto, 2011; Wilson-Mendenhall et al., 2011).

In Experiment 1, context affected RTs but had no significant effect on the accuracy of affective or non-affective judgments of pictures. In Experiment 2, context affected both RTs and accuracy: overall, word judgments that were faster were also more accurate, on average.^2^ Together, these results indicate that the RT effects we observed were not due to a speed-accuracy tradeoff.

Details of both the RTs and the accuracy results suggest that representations activated by target words (in the context of biasing picture judgments) may have been more malleable than the representations activated by target pictures (in the context of biasing word judgments). We obtained RT context effects of similar sizes for scene judgments and for word judgments (i.e., we found 2-way interactions of context type and judgment type, the magnitude of which did not differ statistically between experiments), the details of the scene- and word-judgment data were different descriptively. For the scene targets (Experiment 1), affective judgments were made faster than the non-affective judgments, in both contexts (i.e., there was a main effect of judgment type as well as the predicted interaction). For the word targets, however, we observed a full crossover interaction. Likewise, context affected accuracy significantly for word judgments but not picture judgments. On one possible explanation for these differences between experiments, the mental representations participants activated in response to target scenes (which were detailed color photographs) may have been more constrained by the stimuli themselves than was the case for the representations they activated in response to target words. Whereas words name generic types (e.g., “puppy” can refer to any puppy), pictures depict a specific instance of a type (e.g., a photo must be of a specific puppy). Therefore, representations activated in response to our target photographs may be more constrained than representations activated in response to the target words (cf., Hermans et al., 1994). In addition to the possibility that words tend to cue more variable representations than photographs, it is possible that photographs provided a stronger biasing context than words did, again due to their greater specificity.

## Conclusion

Holding both the stimuli and the judgments constant, the context in which people make affective and non-affective ontological judgments can determine the relative speed of affective and non-affective processing, for both pictures and words. Neither the Affective Primacy Hypothesis nor the Cognitive Primacy hypothesis is uniquely supported when processing context is taken into account. Thus the question of whether affective or non-affective ontological information has processing priority is ill-posed. These findings are consistent with a view of the mind according to which words and pictures activate different neurocognitive representations every time they are processed (see also Schyns, 1998; Elman, 2004; Casasanto and Lupyan, under review), the specifics of which are co-determined by the stimuli themselves and the contexts in which they occur.

## Conflict of Interest Statement

The authors declare that the research was conducted in the absence of any commercial or financial relationships that could be construed as a potential conflict of interest.

## Footnotes

^1^Weaver et al. (2010) used a design similar to ours while examining a debated issue in visual search, i.e., semantic influences from briefly presented peripheral cues. Despite similarities between the studies, Weaver et al.’s goals were different from ours, and their results do not license the same conclusions as ours. One reason is that Weaver et al. did not hold the stimuli for creating the context constant: They used male/female first names for creating the non-affective context, and positively/negatively words for creating the affective context. By contrast with Weaver et al. (2010), the present study held the context stimuli constant. Furthermore, Weaver et al. did not analyze their data in a way that bears on the question of Affective vs. Cognitive primacy (which was not their goal). Visual inspection of their Figure 1 (combining columns across four separate plots) suggests that if they were to reanalyze the data, they would observe a trend toward an effect of the affective/non-affective verbal processing context on affective/non-affective processing of pictures that is compatible with the present findings.

^2^One reviewer pointed out that there appears to be a speed-accuracy tradeoff within affective judgments in Experiment 2. This trend, however, is not statistically reliable according to the more conservative RIS model. It would be inappropriate to interpret this unpredicted, marginal effect, especially in light of the facts that there was no trace of speed-accuracy tradeoff in Experiment 1. Furthermore, there was no speed-accuracy tradeoff in Experiment 2, considering all of the data, combined (and we had no prediction that would license breaking it down). Finally, the opposite pattern is found in the non-affective condition of Experiment 2 (i.e., trials that were more accurate were also faster), and there is no speed-accuracy tradeoff in the data from Experiments 1 and 2, combined.
